# 503. Post-vaccination SARS-CoV-2 Infections and Immune Response in People with Immune Disorders

**DOI:** 10.1093/ofid/ofad500.572

**Published:** 2023-11-27

**Authors:** Mackenzie Zendt, Fausto Bustos Carrillo, Rahul Subramanian, Viviane Callier, Ana Ortega-Villa, Emily Ricotta

**Affiliations:** National Institutes of Health, Bethesda, Maryland; National Institutes of Health, Bethesda, Maryland; National Institutes of Health, Bethesda, Maryland; Frederick National Laboratory for Cancer Research, Bethesda, Maryland; National Institutes of Health, Bethesda, Maryland; National Institute of Allergy and Infectious Diseases, Baltimore, Maryland

## Abstract

**Background:**

While some immune-deficient persons (IDP) face higher risk for severe SARS-CoV-2 infection than the general population, little is known about their immune response to post-vaccination infections.

**Methods:**

A cohort of 217 IDP and 54 healthy volunteers (HV) were followed from April 2021 to April 2023. Blood was collected at baseline, 1-, and 6-months post vaccination. Anti-spike IgG response was assessed by ELISA and the T cell receptor repertoire was sequenced. SARS-CoV-2 infection was actively monitored for 6 months post-vaccination; participants could self-report at any point.

**Results:**

Thirty-six percent of IDP and 46% of healthy volunteers experienced a post-vaccination SARS-CoV-2 infection (p=0.21). Infections occurred from September 3, 2021 to March 25, 2023 and were primarily of the Omicron lineage. Clinical symptoms and severity did not significantly differ between groups, nor did the time from last vaccination to infection (IDP: 140 days, HV: 161 days, p=0.28). While ∼50% of both groups received ≥3 doses, IDP received more doses pre-infection than HV. Indeed, 30 IDP receiving ≥3 monovalent doses and 6 IDP receiving ≥4 monovalent doses were infected (Figure 1). Of the 84 (39%) IDP and 19 (35%) HV who received a bivalent booster, 9 (11%) IDP and 2 (11%) HV later experienced an infection (p=1). Among the 50 IDP who received Evusheld, 9 (18%) experienced an infection after its receipt. Infection increased anti-spike IgG relative to infection-naïve participants 6 months post-dose 2 through post-dose 4 in IDP and 6 months post-doses 2 and 3 in in HV; at later timepoints, IgG levels were the same (Figure 2). The diversity of the T cell repertoire was similar across the cohort; the magnitude of response was lower in IDP and had more variation by disorder.

**Figure 1.**
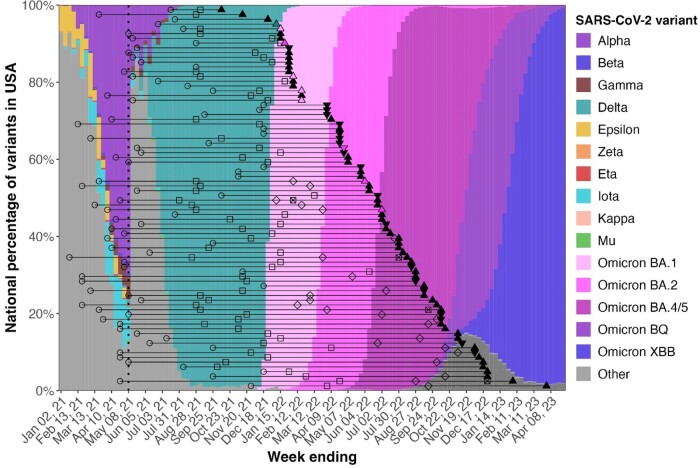
Swimmer plot of breakthrough infections relative to SARS-CoV-2 waves in the US.

The dates of dose 2 (open circle), dose 3 (open square), dose 4 (open diamond), dose 5 (crossed box), and 1st breakthrough infection (triangle) are shown for all 80 infected participants. Upright triangles represent IDP participants (n=60), and upside-down triangles represent HV participants (n=20). The color of the triangle corresponds to the known causative SARS-CoV-2 variant; infections for which the causative variant is unknown are shown in black. The background colors show the national SARS-CoV-2 variant sampling percentages from the CDC. The dotted line indicates the start of the study period, April 29, 2021.

**Figure 2.**
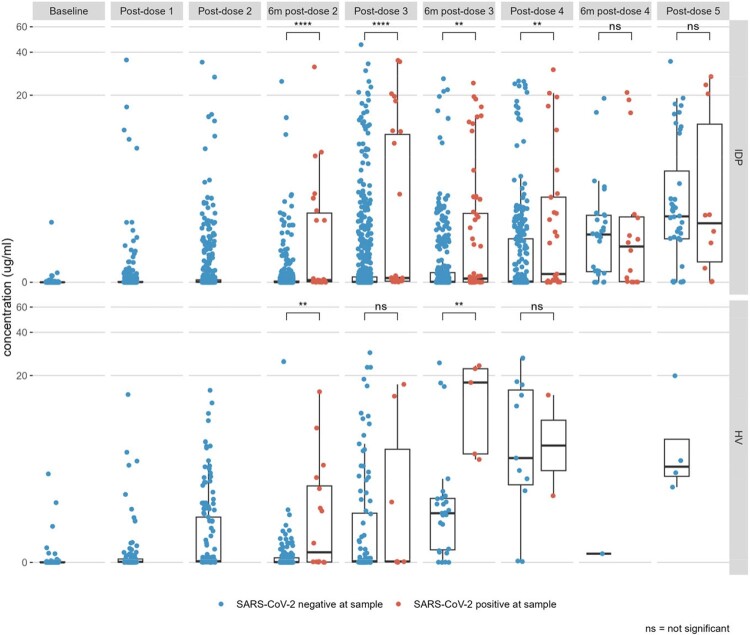
Spike IgG titers by SARS-CoV-2 status at sample

Immune Deficient Persons (IDP) who were SARS-CoV-2 positive had significantly higher titers at multiple timepoints, while Healthy Volunteers (HV) only showed different titers by SARS-CoV-2 status at 6 months post-doses 2 and 3.

**Conclusion:**

Despite reporting high preventative behavior to avoid SARS-CoV-2, IDP experienced post-vaccination infections at a similar rate as HV, mostly during the Omicron period. Less than half of IDP have received a bivalent booster. Additional doses, especially the bivalent dose, enhances the immune response in this population.

This research was supported by the Division of Intramural Research, NIAID, NIH.

**Disclosures:**

**All Authors**: No reported disclosures

